# Introducing adaptive waves as a concept to inform mental models of resilience

**DOI:** 10.1007/s11625-015-0316-6

**Published:** 2015-06-24

**Authors:** Tobias Luthe, Romano Wyss

**Affiliations:** 1 Institute for Tourism and Leisure, University of Applied Sciences HTW Chur, Comercialstrasse 20, 7000 Chur, Switzerland; 2 Chair for Human-Environment Relations, Department of Geography, Ludwig-Maximilians-University Munich, Luisenstrasse 37, 80333 Munich, Germany

**Keywords:** Sustainable society, Resilience planning, Crisis, Deliberate transformation, Governance scales

## Abstract

While ecological resilience is conceptually established, resilience concepts of social–ecological systems (SES) require further development, especially regarding their implementation in society. From the literature, (a) we identify the need for a revised conceptualization of SES resilience to improve its understanding for informing the development of adjusted mental models. (b) We stress the human capacity of social learning, enabling deliberate transformation of SES, for example of SES to higher scales of governance, thereby possibly increasing resilience. (c) We introduce the metaphor of adaptive waves to elucidate the differences between resilience planning and adaptation, by conceptualizing adaptation and transformation as dynamic processes that occur both inadvertently and deliberately in response to both shocks and to gradual changes. In this context, adaptive waves stress the human and social capacity to plan resilience with an intended direction and goal, and to dampen the negative effects of crises while understanding them as opportunities for innovation. (d) We illustrate the adaptive waves’ metaphor with three SES cases from tourism, forestry, and fisheries, where deliberate transformations of the governance structures lead to increased resilience on a higher governance scale. We conclude that conceptual SES resilience communication needs to clarify the role and potential of human and social capital in anticipating change and planning resilience, for example, on different scales of governance. It needs to emphasize the crucial importance of crises for innovation and transformation, relevant for the societal acceptance of crises as drivers of adaptation and transformation. The adaptive waves’ metaphor specifically communicates these aspects and may enhance the societal capacity, understanding, and willingness for planning resilience.

## Introduction

Adaptation to and mitigation of climate change, coping with social-economic change, designing a zero emission and socially fair economy, and establishing a renewable energy society are current examples of pressing societal challenges that include complex, uncertain and coupled social, ecological, and economic factors [e.g., Blythe (2015), Radermacher (2013), Hennicke (2013), Turner (2013)]. Facing a global human population of nine billion by 2050, scientifically and practically feasible solution strategies for adaptive management of natural resources are urgently needed (Godfray et al. 2010; Oki and Kanae 2006; Foley 2005; Crutzen and Stoermer 2000). A loss of biodiversity and a decrease in ecosystem services have occurred due to overexploitation of natural resources and profit maximization in most parts of the world [e.g., Blythe (2015), Mittermeier et al. (2011), Scholz (2011), Ehler (2008), Millennium Ecosystem Assessment (2005)]. This magnitude of human influence led to the coining of the term anthropocene (Crutzen and Stoermer 2000; Steffen et al. 2007), denoting the present time interval in which many geologically significant conditions and processes are profoundly altered by human activities. The three sustainability domains—namely ecosystems, societies, and economies—are interlinked, but still often treated separately when determining the sustainability and resilience of our societies, thereby artificially decoupling ecosystems from social (-economic) systems (Blythe 2015; Xu et al. 2015; Kauffman 2014; Folke 2010; Holling 2001; Walker et al. 2004; Brand and Jax 2007). New approaches are needed to overcome societal obstacles to sustainable development, specifically to increase social adaptive capacity, understanding and willingness to change, and improved risk communication (Lindenfeld et al. 2014; Holdschlag and Ratter 2013; Gallopin 2001). This paper contributes to the sustainability and resilience discussion with the following objectives:

1. Elucidate the metaphor of adaptive waves in order to enhance understanding of the resilience concept in society and to contribute to its application.
2. Add a governance-scale transformation component to the conceptualization of resilience in SES, incorporated in the adaptive waves’ concept.
3. Illustrate the adaptive waves’ concept on three case studies from tourism, forestry, and fisheries, where deliberate governance transformation increases resilience of resource-dependent social systems.

## Societies coping with crises

When trying to understand and to manage transformations toward a more sustainable, resilient society (WBGU 2011; Schneidewind 2013), the term social–ecological systems (SES) is commonly used to describe coupled Human-Environment Systems (HES) [e.g., Xu et al. (2015), Blythe (2015), Holdschlag and Ratter (2013), Toledo et al. (2013), Westley et al. (2013), Scholz (2011), Folke (2010)], including economic and political aspects (Walker et al. 2004). While breakdowns such as the Euro crisis have far-reaching negative effects on key economic and social variables—such as an increasing financial debt and high unemployment rates—a decline in world carbon emissions due to a downturn in economic activities might be regarded as an ecologically beneficial feedback effect from a broader SES perspective. Crises in general are interpreted in the public discussion and the broader public media—who expect linear or progressive (economic) growth—as being unexpected, abnormal, and destructive phenomena [e.g., Lindenfeld et al. (2014), Hampe (2013)]. Natural systems function in cycles of decline and growth. Long-term stability is achieved by repetitive instability; crises are necessary triggers for innovation and to build adaptive capacity [e.g., Lindenfeld et al. (2014), Cumming (2011), Folke (2006), Carpenter et al. (2001)]. While ecological resilience is conceptually established, new methods and tools for conceptualizing resilience of SES have emerged, but their implementation and application require further refinement [e.g., Kofinas et al. (2013)].

Connecting and influencing capacity, understanding, and willingness to transform complex SES toward sustainability are associated with learning about resilience (Xu et al. 2015; Cumming et al. 2005; Gallopin 2001). Effective learning depends on the steady “experimentation in both the virtual and the real worlds, and feedback from both informs the development of mental models” (Sterman 2000, p. 34), which is key variables of change (Holdschlag and Ratter 2013). The currently available metaphors and frameworks of SES resilience, primarily originating from understanding ecosystems, need some elucidation to enhance the development of new, more specified conceptualizations of SES resilience [e.g., Xu et al. (2015), Lindenfeld et al. (2014), Folke (2010)]. These may then inform the development of mental models, understood as “assemblies of fragmentary beliefs”, which people “will then use to reach their conclusions” (Morgan et al. 2001, p. 21), e.g., to judge what aspects “in a complicated situation are worthy of attention” (Morgan et al. 2001, p. 21).

Available SES resilience frameworks need to better communicate social specifications of the resilience concept—in particular, social agency, the role of social networks, diverse and uncertain knowledge systems, mental models of risk and environment—and the constructive coping with crises and risk, more generally, to a broader audience (Xu et al. 2015; Lindenfeld et al. 2014). Enhancing the provision and development of metaphors, concepts, frameworks, and mental models to understand and learn about resilience will help to develop capacity, understanding, and willingness to engage toward sustainability (Xu et al. 2015; Lindenfeld et al. 2014; Morgan et al. 2001).

## Resilience of ecosystems versus social–ecological systems

Resilience of ecosystems can be understood as “the capacity of a system to absorb disturbance and reorganize while undergoing change so as to still retain essentially the same function, structure, identity, and feedbacks” (Walker et al. 2004, p. 6). The adaptive cycle concept (Gunderson and Holling 2001) explains the adaptation of ecosystems in the form of a cycle of the four ecosystem functions: exploitation, conservation, release, and reorganization. Peaks of evolution (climaxes) take place in the fast transition phase between release (*Ω*) and reorganization (*α*) with high levels of flexibility, while the transition from exploitation or growth (*r*) to conservation (*K*) is characterized by the slow accumulation of resources with high levels of stability (Brand and Jax 2007; Gunderson and Holling 2001). The adaptive and evolutionary nature of adaptive cycles is that they are organized in a nested, dynamic, and adaptive set in space and time, the panarchy (Holling et al. 2001). Adaptive capacity of natural systems is described by two panarchy connections: the ‘revolt’ function stands for a cascading effect where fast and small events trigger a change in larger and slower cycles; and the ‘remember’ function draws upon the maturity and potential of larger and slower systems in their conservation phase (Gunderson and Holling 2001). The ‘remember’ function is described as a process in which it seems that the panarchy connection draws upon the “accumulated wisdom and experiences of maturity” of systems that have undergone crises before (Gunderson and Holling 2001), which can be understood as a form of unintended learning. Such systems recover from shocks, e.g., by means of natural selection in times of ecological crisis (see the original resilience concept in Holling 1973, as well as Cumming 2011).

Resilience of social organizations as discussed by Linnenluecke et al. (2012) is the continuing capacity to recover from disturbances as well as the capacity to rebound from adversity in a strengthened and more resourceful way. A broad body of literature [e.g., Holdschlag and Ratter (2013), Folke et al. (2004), Olsson et al. (2004), Gunderson (2000), Hughes et al. (2005)] focuses on the possibility of organizations and individual actors to (co-) manage ecosystems in order to allow societies and economies to cope with ecosystem changes and the changing provision of ecosystem services [e.g., Adger et al. (2005)]. Following Folke et al. (2010), one can distinguish between a specified and a general (SES) resilience perspective. While specified resilience deals with questions of a system’s (or part of a system’s) resilience to specific impact factors, general resilience describes the system´s capacity to deal with various forms of change, potentially at different moments in time (Elmqvist 2014; Carpenter et al. 2012). When applied to the planning of social–ecological resilience and the deliberate initiation of transformation processes, specified resilience understandings are predominant (Folke et al. 2010). While fundamental changes to SES can be driven both by social actors from within the systems as well as by external pressure factors and shocks [see e.g., Walker et al. (2004)], deliberate transformations within SES tend to be initiated on lower (governance) scales and with respect to specific resilience issues (Folke et al. 2010). Pelling and Manuel-Navarrete (2011) point to the central importance of social capital and the agency potential of social actors in planning and implementing transformative processes. Resilience of SES is the result of both the structural properties of a SES (e.g., networks of social–ecological interdependencies), as well as the action-potential of individuals (human capital), groups, and communities (social capital), able to drive adaptation and transformation processes on different scales (Xu et al. 2015; Wyss et al. 2014; Holdschlag and Ratter 2013; Pelling and Manuel-Navarrete 2011). In this line of thought, resilience of SES is related to coping with external stresses by maintaining the stability of the social structures, while ensuring the flexibility and diversity necessary for innovation and development in the broader context of adaptation and transformation [e.g., Xu et al. (2015), Garmestani and Benson (2013), Westley (2011), Nelson et al. (2007)]. The directed and planned recovery from shocks in a strengthened way, and the capacity for anticipation of crises by social learning are key differences in the resilience of social systems when compared to natural systems (Folke 2006).

The adaptive cycle and panarchy concepts of resilience have been widely applied in the discussion of resilience in SES [e.g., Holling (2001)], among others because of a lack of alternative concepts. A direct application of an ecological resilience concept to SES will lead to conceptual and normative difficulties [e.g., Luthe et al. (2012), Adger (2000), Duit and Galaz (2008), Gunderson and Holling (2001)], since existing models and heuristic conceptual frameworks focus mostly on large-scale disturbances or on accumulations of minor disturbances (Vogus and Sutcliffe 2007), and they mostly neglect the capacity of social actors to learn from prior experiences due to their forward-looking behaviors (Westley et al. 2001).

Holling et al. (2001) acknowledge that the use of the panarchy concept for social systems is an abstraction and that its original application to ecosystems has been challenged by social scientists. Gunderson and Holling (2001) describe social systems as variations or departures from the adaptive cycle, “incorporating foresight and adaptive methods that stabilize variability and exploit opportunity” (p. 62). In contrast to species in a pure ecosystem context, social actors within SES have the capacity to learn and deliberately transform the governance structure of a SES in a previously planned direction to dampen or mitigate shocks and crises (Folke 2006). Related to this, Holling (2001, p. 401) identifies three features distinguishing human from ecosystems: foresight and intentionality, communication of ideas and experiences, and the use of technology. A SES resilience concept should thus capitalize more on the capacity for ‘constructive’ (=deliberate) transformation on different social and governance scales (Kofinas et al. 2013; Olsson et al. 2010; Folke et al. 2009). More recently applied frameworks, such as Nelson et al. (2007), Smit et al. (2010), Hovelsrud and Smit (2010), and Lovecraft and Eicken (2011), however, include the social capacity to anticipate and deliberately plan for future changes, but lack the integration of time, the capacity to dampen the strengths of crises, and the capacity for governance intervention (i.e., for the transformation to another governance structure or scale) in one unified and easy-to-grasp framework, as we will outline in the following.

## Inadvertent and deliberate adaptive and transformative capacity

Both ecological and social-economic systems are forced to adapt to short-term or sudden changes, as well as to long-term or gradual changes. Thereby, adaptability or adaptive capacity, according to Walker et al. (2004), describes the capacity of the (social) actors within a system to manage resilience, in other words, to influence the flexibility and stability of a SES in the face of external challenges. In the context of climate change, Moser and Ekstrom (2010, p. 22026) in a slightly different perspective define adaptation as “changes in social–ecological systems in response to actual and expected impacts [of climate change] in the context of interacting non-climatic changes. Adaptation processes to changing environmental, social and economic conditions require actions and initiatives by various actors from different backgrounds, and on different scales of action. Adaptation (…) can range from short-term coping to longer term, deeper transformation.” In contrast to the established adaptive cycles concept in its original function as a metaphor to classify ecosystems (Holling 1973, 2001), adaptive actions in SES include a clear development in time steered by social learning and innovation, and based on experiences and anticipation, possibly leading to transformation processes (Lindenfeld et al. 2014; Holdschlag and Ratter 2013).

Transformation in this context is seen as the capacity of a system to change its properties and meaning, “to fundamentally alter[ing] the nature of a system” (Walker et al. 2004, p. 6). Transformation allows the actors of a system to break out of given development paths and undesired, yet stable situations [lock-in effect, see e.g., Hassink (2010), Allison and Hobbs (2004)], restricting adaptation and adaptive processes. Following O’Brien (2012), there are two distinct forms of transformation—deliberate transformation, meaning transformation with the intent of achieving a certain goal, and inadvertent transformation as the unintended consequence of a (adaptation) process or event. This distinction also reverberates in the separation between emergent transformation and purposive transitions, as proposed by Smith et al. (2005). Transformation is always closely linked to individual adaptation measures and goals, but aims further and leads to more fundamental structural changes in the system (Nelson et al. 2007). For the remainder of the paper, we understand deliberate transformation as a social process of learning and innovation with a specifically set goal that takes place in a SES such as a resource-dependent community, but not in ecosystems.

**Fig. 1:**
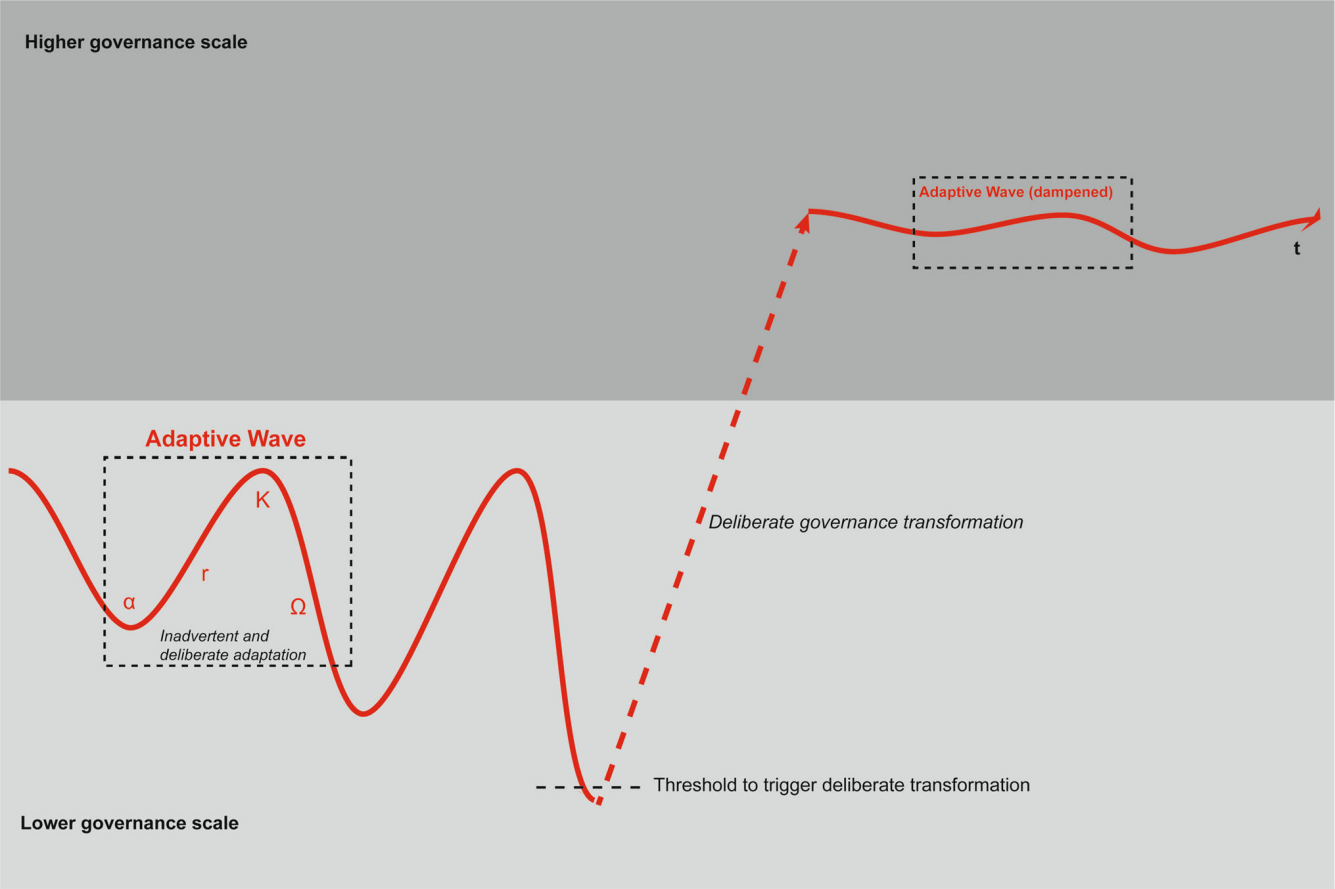
The adaptive waves’ metaphorical concept of SES resilience

Deliberate transformation has a set and planned transformation goal, while inadvertent transformation is unplanned and either the consequence of a number of incremental adaptation steps, or the outcome of a random process (Fig. 1). SES have the capacity for inadvertent and deliberate transformation due to social and economic action, whereby deliberate transformation processes are closely linked to agency and social learning, which allows actors to avoid to reiterate developments of the past that may lead to negative consequences, provided that suitable governance structures support such transformation [e.g., Lindenfeld et al. (2014), Holdschlag and Ratter (2013)]. In this context, our understanding of steering deliberate transformation processes is linked to the transitions management ideas proposed by Rotmans and Fischer-Kowalski (2009) and Martens and Rotmans (2005). They understand transitions as a serious of connected changes and possible development paths during which humans are able to adapt to, learn from and anticipate new situations. The direction, size, and speed can be influenced through policy and specific measures and circumstances. The anticipated steering capacity on a timely development path is similar in our adaptive waves’ deliberate transformation idea, though our metaphor places an emphasis on scale issues and the effect of crises. Deliberate transformation processes are often initiated and sustained with a specific goal in mind, giving the transformation process a specific socio-political anchoring and purpose (O’Brien 2012; Berkhout 2002). They are often at the basis of broader change in SES. Their initiation can, in many cases, be traced back to a smaller group of people, or specific social pressure groups (O’Brien 2012; Pelling et al. 2008; Olsson et al. 2006). Social learning is one possibility of how novel approaches and ideas can spread within specific communities of practice [see e.g., Nykvist (2014)]. As Cumming et al. (2013) point out, social learning must be embedded within adaptive governance initiatives in order to achieve broader social acceptance and ultimately drive deliberate transformation processes, allowing for multiple cultural, cognitive, institutional, and political barriers to be overcome [see also Pelling and Manuel-Navarrete (2011), Moser and Ekstrom (2010)].

## Scale-dependent governance of resilience in SES

A governance structure supporting resilience of SES has to meet two fundamental criteria, according to the literature [e.g., Ernstson et al. (2010), Manring (2007), Folke et al. (2005)]: (1) Preparing for disturbance by creating and maintaining diversity to prepare for change (by enhancing decentralized processes of social learning) and (2) responding to disturbance by creating and maintaining flexibility and the capacity to steer more centralized forms of collective action. The creation and maintenance of diversity and flexibility are dependent on the collective social capital and on the individual human capital (Coleman 1988; Schuller 2001).

The flexibility of a system allows for the implementation of short-term adaptation processes to external and internal challenges. In contrast to short-term shocks, long-term adaptation to more subtle changes implies learning processes and innovation, and coordination of such collective action. Thus, smaller systems on lower governance scales may be more resilient to shocks and quicker changes through more flexibility and short-term adaptation, while larger systems on higher scales of governance may react slower to fast changes, but they may be better capable of organizing collective action from a more diverse pool of nodes and ties, and prepare for transforming the system (Young 2002). Following the argumentation in Walker et al. (2004), SES tend to lose resilience at smaller scales, although they are more manageable at such scales: for example, a patch of land is easier to manage than a whole landscape, but is at the same time less resilient to external impacts. Within the same line of thought, Holling (2001) points to the fact that on a higher scale of organization, changes take place at a lower speed and over bigger areas, while changes on a lower scale can initiate adaptive processes on a higher scale, and changes on a higher scale can reciprocally also influence adaptive and transformative processes on a lower scale.

Social networks play an important role in steering governance processes on different political, juridical, or geographical scales, e.g., with regard to ecosystem management, as well as for processes of social learning within specific institutional settings (Kauffman and Arico 2014; Holdschlag and Ratter 2013; Manring 2007; Olsson et al. 2007; Folke et al. 2005). The success of governance and management processes in shifting between flexibility and diversity depends significantly upon the existence and strength of social ties between actors and institutions, especially when looking at actor-groups and institutions that are engaged on different scales of governance. Scale-crossing connections are often controlled by a small number of actors the literature refers to as brokers [e.g., Ernstson et al. (2010)]. They are of high relevance to understand how information flows and collaboration across scales take place, influencing both the management of ecosystems as well as broader governance processes in the case study regions (Duit and Galaz 2008; Sørensen and Torfing 2003). Individual actor-groups often have access to scale-specific information about the system, which for an efficient attribution of access to ecosystem services is to be shared over scales (Olsson et al. 2007; Ashby 2003; Bandura 1977).

The main goal in building resilience of a SES is to achieve long-term functionality and stability of the system, a stability of the social structure that is dynamically driven by switching between flexibility (for responding to change) and diversity (for preparing to change) (Folke et al. 2005). This understanding of stability in a network governance context can also be related to the adaptive cycle concept following Holling (1973, 2001): the adaptive cycle phases of exploitation and conservation are characterized by high levels of stability (and low levels of flexibility), where resources are slowly accumulated and transformed, whereas the phases of release and reorganization are characterized by high levels of flexibility (and low stability) with opportunities for innovation.

## Proposing the metaphor of adaptive waves

In order to conceptualize and illustrate the discussed features of SES resilience in a more clarified and specified way that may support social learning, the development of adjusted mental models, and ultimately enhance societal adoption and implementation of the resilience concept, we propose the metaphor of adaptive waves. Given the capacity for social learning to anticipate crises, for directed governance on different scales, and the timeline and goal of sustainable development in SES, adaptive waves integrate the adaptive cycle phases exploitation or growth (*r*), conservation (*K*), release (*Ω*), and reorganization (*α*) as the effects of the processes inherent in the different cycles on a time line and direction of development (Fig. 1). The varying oscillation of the adaptive waves’ results from the changing behavior of their underlying state variables, but the social component within an adaptive wave may as well influence their oscillations based on inadvertent or deliberate adaptive action. Series of adaptive waves continuously alters with variable oscillation and amplitude, duration, and speed. The waves clearly indicate a timeline and a direction of development, with either inadvertent (unintended) or deliberate (intended), fast or slow adaptation, functioning as parts of each adaptive wave on different scales of governance. The adaptive waves clarify a main difference in resilience of SES in comparison to ecosystems: while in ecosystems, different states of equilibria are to be gained or re-gained after shock by random, selection-on-diversity or fed-back from maturity-based processes of adaptation and transformation (Westley et al. 2001; Levin 1999), adaptation and transformation in SES can be a deliberate process of directed, planned development with a clear goal [e.g., of a sustainable society, see Cumming et al. (2013), Folke (2006)]. This could be a transformation of the SES onto a higher governance scale (Fig. 1). Such a process builds upon the human and social capital to anticipate, to learn, and to organize SES governance on different scales (Westley et al. 2001), informed by mental models as assemblies of fragmented information to prioritize action in complex situations (Morgan et al. 2001). Both adaptation to fast changes (shocks) and anticipated transformation to gradual changes happen constantly within the adaptive waves and evolve over time as indicated by the sets of adaptive waves and their varying oscillation. If—as a consequence of shocks or maladaptation—phases from conservation to release repeatedly occur or are strong enough to reach a (social) threshold (Xu et al. 2015), the SES governance may deliberately get transformed onto a higher organizational level or governance scale (Fig. 1). On a higher governance scale, resilience may be higher if the advantages of e.g., faster action on a lower governance scale are maintained and well integrated (Luthe and Wyss 2015; Walker et al. 2004). This is illustrated by the dampened oscillation of the adaptive waves, where phases of decline in the release phase following crises are less severe and shorter (Fig. 1). Minor crises are part of the oscillating waves and drive adaptation processes, while major crises can lead to scale-crossing transformations, if the oscillation due to the shocks linked to the crises is strong enough to cross a threshold of action. Deliberate transformation can also be stimulated by long-term gradual changes through anticipated learning and planned change of the SES governance, but it remains a process requiring deliberate human decisions, unlike random evolutionary processes in ecosystems.

Resilience of a SES is thus conceptualized as a dynamic process of a repetitive series of adaptive waves with altering phases of flexibility and stability, allowing for quick adaptation to short-term changes expressed in the varying oscillation of the waves. Diversity supports social learning and innovation, fundamental for transformation in the context of more structural and planned adaptive action. Both types of change require either fast, quick responses or long-term strategies of innovation and transformation. Fast, sudden changes mostly relate to clearly identifiable tasks, while slow, gradual change—such as climate change—mostly relate to more complex, not clearly identifiable adaptation tasks. An increase in overall resilience would mean to not prevent phases of exploitation and decline, but to reduce the oscillation of the adaptive waves and thus lower the amplitude and extent of system breakdowns and dampen the effects of crises. Coping with crises as windows of opportunity on a path that can be designed toward a specific goal is relevant for societal learning toward sustainability. Conceptualizing and illustrating these processes can help the formation of mental models to ultimately spur and support the capacity, understanding, and willingness to change in society.

Summarizing the adaptive waves’ concept offers a clarified and specified conceptual understanding of SES resilience that may enhance societal learning and action. The adaptive waves concept explicitly incorporates (a) governance scale issues, (b) a path-dependent time component, (c) a clear development goal, (d) the possibility of dampening (but not eliminating) the impacts of undesirable external influences in SES by governance intervention and understanding crisis as opportunities, and (e) it distinguishes between inadvertent and deliberate transformations. In the following paragraph, we illustrate this metaphor of adaptive waves by means of three SES.

## Illustration of adaptive waves to different SES

We first describe a tourism-dependent community SES in the Alps and discuss it in detail as an illustration of the adaptive waves’ concept. We then summarize two further cases on reforestation in the Nigerian Sahel desert and on reducing illegal fishing in the Southern Ocean to strengthen our following conclusion.

### Governing alpine communities with tourism-based economies on a regional scale

Alpine tourism destinations are examples of complex, natural resource-based SES on a regional level, dependent on specific weather conditions and snow, as well as on favorable socio-economic circumstances. Both the natural as well as the social-economic variables undergo natural oscillations, e.g., in short-term weather variability, long-term climate trends, and in terms of tourist numbers and economic revenues. Based on empirical work in the Swiss Gotthard tourism region as a real-world SES, we assess and compare resilience of three Alpine communities and their inclusion in a DMO (destination management organization) by quantitative and qualitative network analysis (Luthe and Wyss 2014). Discussing published results from this case (Luthe et al. 2012; Luthe and Wyss 2015), we exemplify how the understanding of adaptive waves can help to conceptualize the capacity of SES for planning resilience and how deliberate transformation may allow to build resilience on a higher governance scale.

**Fig. 2:**
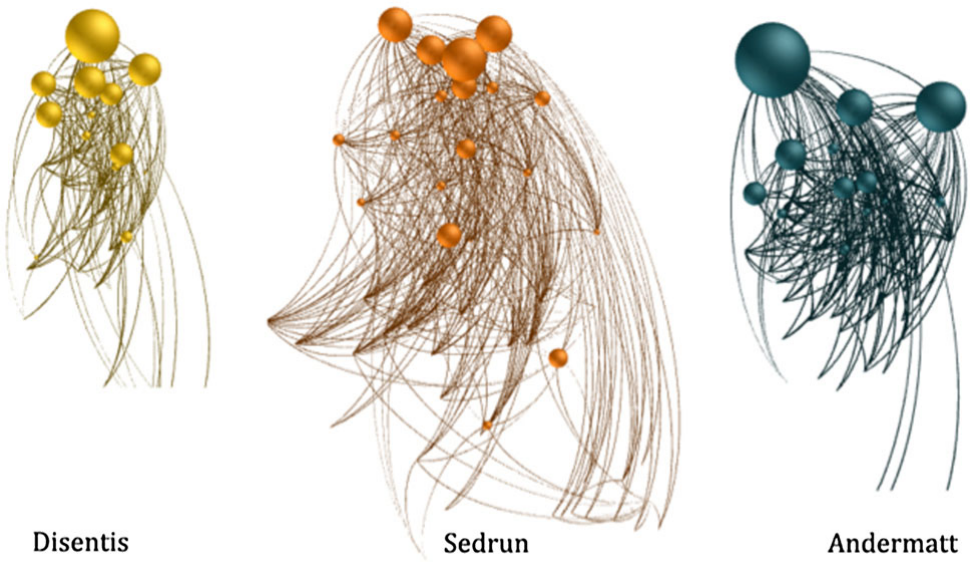
The tourism networks of the three communities Disentis, Sedrun, and Andermatt. Nodes are the tourism supply chain businesses, links are the indicated collaborations, node sizes indicate the importance of the single actors in connecting others (adopted from Luthe and Wyss 2015)

The Gotthard tourism system comprises collaborating businesses along the tourism supply chain within each of the three main communities: Andermatt, Sedrun, and Disentis. Figure 2 shows the collaborative tourism business networks of these three communities in a force directed layout (Kobourov 2012), where the size of the individual nodes indicates their importance (betweenness centrality) in connecting others. The local tourism businesses generate most of their revenue in winter, and are highly vulnerable to climate and further social-economic change. Individual supply side tourism actors are embedded in destination governance structures. The individual businesses contribute a certain share of their turnover to a community or regional-based DMO. In return, the DMO takes over tasks such as marketing toward outside markets, cross-company product developments or the defense of political interests, which call for cooperative efforts and which are prone to coordination and free-riding problems (Raich and Pechlaner 2006; Beaumont and Dredge 2010). This means that individual economic actors can adapt to changing conditions by various forms of social learning, e.g., by product or process innovations as offering a new product (variety or diversification), by teaming up to create new economic structures to bring products to the market (and ultimately to customers), or by pressing for joint political decisions altering the institutional and regulatory framework within which individual or collective actions take place [e.g., Roman et al. (2010)] both on an individual business scale and on a (joint) community or regional DMO scale.

The economic development of the tourism system on a community governance scale is closely coupled with the short-term variability and long-term trend of the regional and local climate. The communities in the Gotthard region have a snow-based economy linked to their dependency on ski tourists during the winter half-year. Climate change has led to shorter, warmer, and lesser predictable snow coverage, and seasons with little snow cause severe economic problems in these communities. Less snow in winter leads to a decrease in tourist numbers, and this subsequently leads to economic stress in ski areas, hotels, and restaurants. These single mechanisms are interconnected and take the system into a phase of release and reorganization where opportunities for innovation are created. The system may get weakened or even partly break down. Based on the social and human capital present, the system might get out of this phase of crisis to survive and reorganize.

**Fig. 3:**
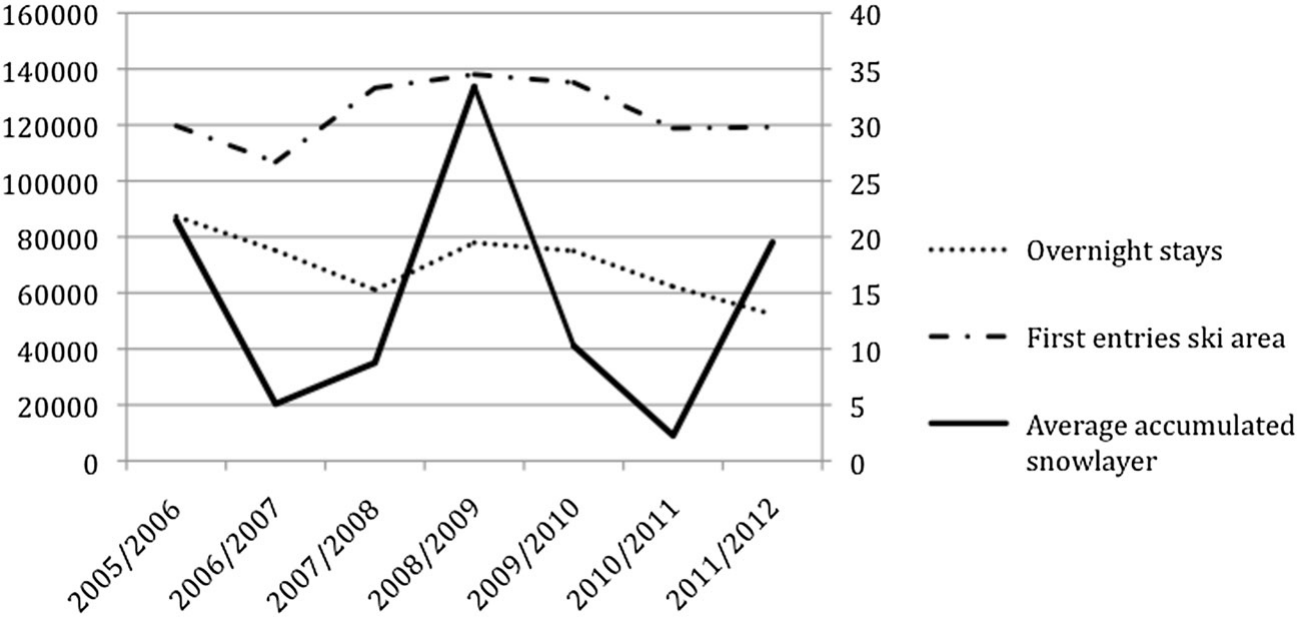
Coupled oscillation of climate and economic data of Disentis for the winter seasons 2005/2006 to 2011/2012 (MeteoSwiss 2013; Bergbahnen Disentis 2012)

Since SES have the capacity to establish different forms of governance on different scales, deliberate transformation can increase resilience in order to dampen the impacts of crises, leading to lower oscillation of the adaptive waves (based on their underlying behavior), while the complete prevention of crises does not seem possible. Figure 3 demonstrates the functional relation of the coupled natural and the social-economic variables from a conceptual angle: the economic success of the community Disentis in terms of overnight stays and first entries of tourists in the ski area is related to the variability in snow coverage, depending once again on the variability of temperature and precipitation. The variability in first entries and overnight stays follows the snow coverage, indicating the coupling of the two variables in the form of two oscillating curves. Based on the available data, the winter seasons 2006/2007 and 2010/2011 were the driest and warmest on records (Beniston 2007; Uhlmann et al. 2009; Falk 2010), while the winter 2008/2009 had more than 90 % more average accumulated snow as compared to the average for the Disentis observation station at 1198 m asl (MeteoSwiss 2013). The first entries of skiers in the ski area as an indicator of broader tourism-based economic activities are directly dependent on the snow coverage and show a coupled variability with 32,000 entries less in 2006/2007 than in 2010/2011 (Fig. 2). With a day ski ticket price of about 55CHF in 2013, this relates to a loss of about 1.7 million CHF for the cableways in terms of cash flow. Overnight stays as a second economic indicator also seem related to snow coverage, albeit with a delay of one season. The delay in the correspondence between overnight stays and snow coverage is most likely due to longer planned booking periods.

After a year with little snow, bookings for the following year dropped. While other factors may influence variability in the economic data as well, the numbers illustrate the causal relation between ecological and social-economic factors in the Disentis tourism case.

**Fig. 4:**
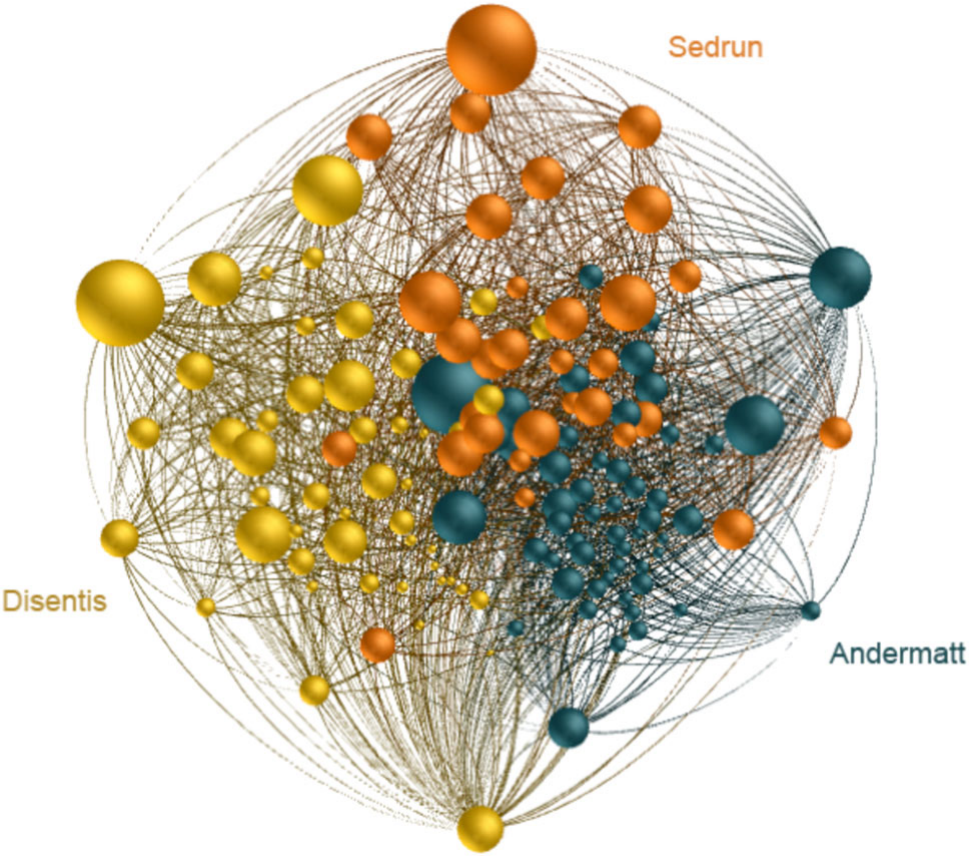
The Gotthard regional DMO (Destination Management Organization) network (force directed layout) with its three interconnected components, the communities Andermatt, Sedrun, and Disentis

Referring to the case of the broader Swiss Gotthard region, the three tourism-dependent communities Disentis, Sedrun, and Andermatt (Fig. 2) will presumably be combined in a new Gotthard DMO on a regional governance scale; the collaborative intra-community networks of the tourism businesses, and public actors from each community will then form a larger network with new inter-community ties (Fig. 4). The step from the community governance scale to the regional governance scale is a form of deliberate transformation with the goal of strengthening economic success and resilience - a common strategy in the tourism industry (Bieger 2008). The construction of a DMO on a higher governance scale is a fundamental alteration of the system and thus a governance transformation (Walker et al. 2004). New governance structures need to be established that alter the way processes—related to tourism management, marketing, adaptation, finances, and administration—are organized and implemented within the communities (Beritelli and Bieger 2014; Pechlaner et al. 2012; Bieger 2008). While the DMO as a coordinating entity is likely to ignite and coordinate processes of social learning, the actual innovation and adaptation steps are to take place through the interaction of the various actors located in the three communities.

Luthe and Wyss (2015) found that both quantitative network metrics and qualitative data validating social processes confirm the higher resilience of the Gotthard tourism system on the regional DMO scale. Figure 4 shows the Gotthard DMO as an ordered network of the collaborating tourism business stakeholders with intra- and—on this higher regional governance scale—inter-community ties. This regional network has a higher resilience by combining advantages of more centralized steering and better community- and core-periphery integration for innovation and adaptation to gradual changes, compared to the individual communities. The community networks on the (lower) community governance scale, on the other hand, have a higher capacity for quick reaction to short-term shocks (Luthe and Wyss 2015). Overall, deliberate transformation as a directed and intended transformation process onto the higher, regional DMO governance scale increases resilience by dampening the oscillation of the underlying behavior of the state variables displayed as adaptive waves.

### Regreening regions in the Sahel of Niger

Sendzimir et al. (2011) report on the case of the Nigerian Sahel zone where a deliberate transformation of the governance system onto a higher scale after re-occurring stresses led to an increase of resilience. Vulnerability of societies and ecosystems of the Nigerian Sahel to climatic and economic changes increased in the late twentieth century due to severe periods of drought leading to famine, massive livestock losses, and human migration (Sendzimir et al. 2011). Outbreaks of drought, famine, and locusts are coupled with a strong increase in population and largely varying annual rainfalls. Figure 2 in Sendzimir et al. (2011) indicates the wavy alterations in annual rainfall and the occurrence of different types of crises, leading to further deforestation and the advancement of the Sahara desert southwards, until a sudden change led to the reforestation of 5 million hectares in two regions of Niger. No single actor, practice, or policy could stop the deforestation, but a network of actors and institutions on different levels, times, and scales initiated this shift in increasing social–ecological resilience. The major driver for the shift in forest decline was a deliberate governance transformation: The International Fund for Agricultural Development (IFAD) supported the establishment of management committees in involved villages which empowered people to experiment and implement farmer managed natural regeneration (Sendzimir et al. 2011). The repeating crises in this case reached a threshold were a deliberate transformation onto a higher governance scale—from the individual actor to a community scale—initiated and amplified adaptive processes on the lower governance scale and helped to increase overall resilience in the region.

### Reducing fishing in the Southern Ocean

Illegal, unregulated and unreported (IUU) fishing is among the biggest threats globally to sustain marine ecosystems and fish stock (United Nations 2011). Oesterblom et al. (2015) describe the practice of large-scale illegal fishing of big fish—mainly Patagonian toothfish—by longline hauling in the sub-Antarctic Seas, leading close to its extinction. Overexploitation by IUU fishing represents an international SES-crisis situation, and in 1997 the full extent of IUU fishing became evident to the Commission for the Conservation of Antarctic Marine Living Resources (CCAMLR). A period of reduced fishing due to actions of various NGOs was followed by a second crisis situation in 2003 with IUU fishing reaching another peak accompanied by a more professional and international organization of such practices. A third crisis occurred in the late 2000s when new net fishing techniques were introduced to IUU fishing, with which the source of catches was harder to derive. The CCAMLR became increasingly concerned about the effects of IUU fishing, while the apparent effects of the described governance crises brought about by new fishery practices offered opportunities for different stakeholder groups—NGOs, the licensed fishing industry, and state agencies, allowing them to implement new practices and policies that were difficult to be introduced in non-crises situations. Repetitive crises created the threshold to deliberately form a new alliance on a higher governance scale. The newly developed tools, such as electronic catch documentation and a rigid tracking system, made collaboration easier and were increasingly used by different state agencies and NGOs. CCAMLR was empowered to function as a brokering node to facilitate collaboration between the NGOs and states with different agendas, but with the common goal to combat IUU fishing. As a result of this deliberate governance transformation, IUU considerably decreased over the last years with an increase in resilience of the marine ecosystem.

All described SES cases, where deliberate transformations followed repetitive and increased crises, illustrate the adaptive wave concept. General adaptive processes—inadvertent and deliberate—function independently on different governance scales as part of repetitive adaptive waves, whereas overall resilience may increase on a higher governance scale. This is partly due to better steering of collective action, partly due to better integration and empowerment of peripheral actors—who provide new ideas—with more central and brokering actors, who are crucial for implementing concrete measures derived from these new ideas. At the same time, the faster communication of the lower governance scale networks can ideally be maintained. Nevertheless, the coupling of social systems with ecosystems will still entail waves of decline and de-growth, necessary to innovate and reorganize, but their oscillation can be dampened.

## Conclusions

In order to transform our societies to become more resilient and sustainable, the understanding of change processes within complex, natural resource-dependent SES (social–ecological systems) needs further elucidation. By proposing the adaptive waves metaphor, this paper helps to clarify how society can cope with change and plan resilience as a deliberate process. Transformation in social–ecological systems, involving, e.g., a deliberate change in governance systems, requires the adjustment of mental models. The adaptive waves’ concept is informed by the adaptive cycle panarchy framework, but offers an original angle to understanding and communicating how society can cope with change. The adaptive waves indicate the constant and dynamic process(es) of social adaptation and transformation to both shocks and gradual developments on a path-dependent time line. The concept clearly states a governance scale component, while facilitating the understanding of the capacity of social learning and anticipation within SES, potentially leading to the dampening of necessarily occurring phases of decline or crises. Adaptive waves enhance an easier understanding of resilience of SES as a path with the objective of developing sustainability in a constantly evolving process over time and with a clear goal of increasing resilience by social learning and deliberate transformations. In the three cases discussed above, different SES show higher resilience following a deliberate (governance) transformation process onto a higher governance scale.

Overall, the social–ecological resilience discussion should take phases of (social) decline and crises more into account as normal and even as constructive in a public understanding, necessary to innovate and to increase resilience, which may be organized on a higher governance scale. Crises can be better understood as unavoidable and thus be accepted as part of the development path of any SES. Crises can be further seen as triggers and drivers of adaptation and transformation processes that will lead to new prosperity, social–ecological wellbeing, and higher resilience. If decline and crises are accepted as unavoidable but constructive, social actors may more likely be motivated to engage in resilience thinking and planning processes to strengthen resilience. Crises and the awareness of their unavoidable occurrence are important triggers to break up, prevent, or at least control phases of social stagnation with low levels of innovation (in the conservation phase), but the impacts of crises can be controlled—reduced oscillation of waves and dampened effects of crises in the release phase—by deliberate transformations of, e.g., the governance system.

Adaptive waves as a metaphor for practical resilience conceptualization allow for better understanding and communicating the process of social learning and transformation as a constant, dynamic, and intended process on a timeline. Social learning and transformation following shocks as well as gradual negative developments are capable of lifting the SES onto a higher resilience level, instantly or over time, reducing oscillation and lowering the amplitude of the adaptive waves while dampening and buffering negative effects in phases of decline and crises. However, decline and crises remain integral and necessary components of resilience and sustainable development in SES to trigger innovation. The adaptive waves’ metaphor helps to inform the development of mental models of SES resilience in order to spur decision-making and action toward the sustainable transformation of our societies.
